# Supplementary material for: Therapeutic B-cell depletion reverses progression of Alzheimer’s disease
Source: Nat Commun. 2021 Apr 12;12:2185. doi: 10.1038/s41467-021-22479-4 (PMC8042032; doi:10.1038/s41467-021-22479-4)
Supplement: Supplementary file 1 — Supplementary Information [file 41467_2021_22479_MOESM1_ESM.pdf]

Supplementary Information

Therapeutic B-cell Depletion Reverses Progression of Alzheimer's Disease

Ki Kim, Xin Wang, Emeline Ragonnaud, Monica Bodogai, Tomer Illouz, Marisa DeLuca, Ross A. McDevitt, Fedor Gusev, Eitan Okun, Evgeny Rogaev, and Arya Biragyn

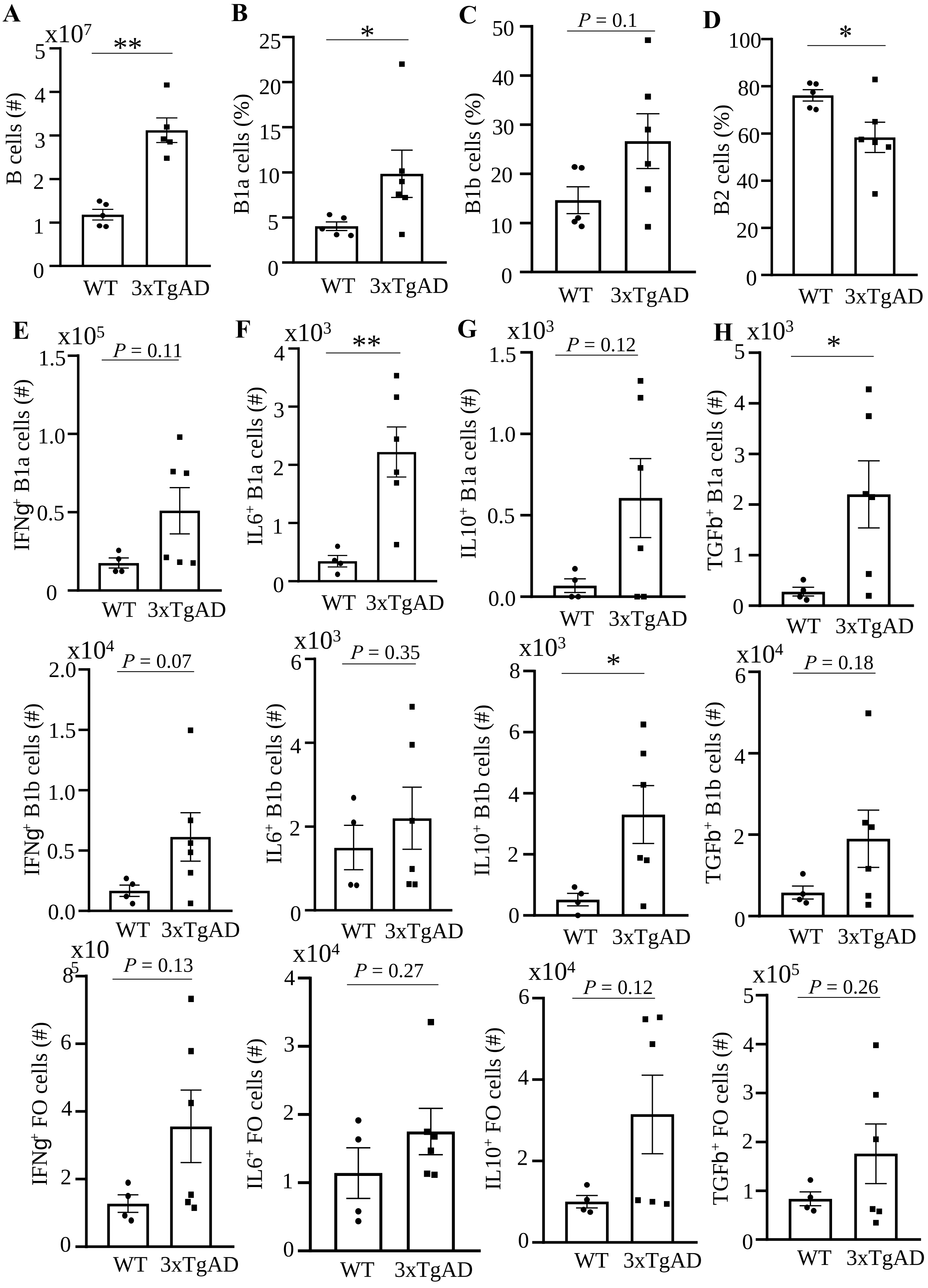
A
B
C
D
E
F
G
H
x10^7
**
*
P = 0.1
*
B cells (#)
B1a cells (%)
B1b cells (%)
B2 cells (%)
WT
3xTgAD
x10^5
P = 0.11
x10^3
**
P = 0.12
IFNg+ B1a cells (#)
IL6+ B1a cells (#)
IL10+ B1a cells (#)
TGFb+ B1a cells (#)
x10^4
P = 0.07
P = 0.35
P = 0.18
IFNg+ B1b cells (#)
IL6+ B1b cells (#)
IL10+ B1b cells (#)
TGFb+ B1b cells (#)
P = 0.13
P = 0.27
P = 0.26
IFNg+ FO cells (#)
IL6+ FO cells (#)
IL10+ FO cells (#)
TGFb+ FO cells (#)

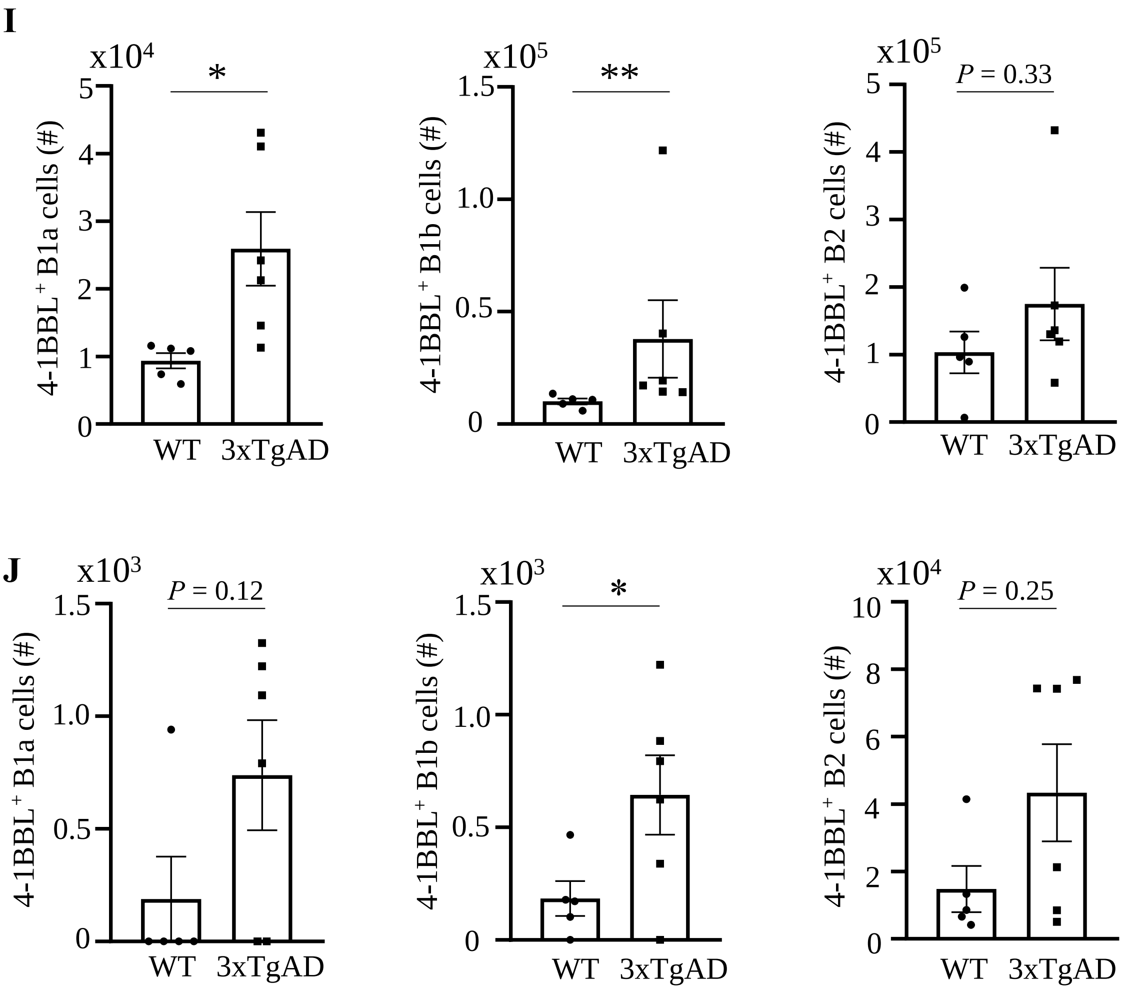

Supplementary Figure 1. 3xTgAD mice increase activated B1 cells. Compared with congenic, age- and sex-matched WT mice, 3xTgAD mice increased $CD19^+$ B cells (**A**) and innate B1 cells (B1a, $CD5^+CD11b^+CD19^+$; and B1b cells, $CD5^-CD11b^+CD19^+$ in CLN, **B** and **C**) but decreased conventional B2 cells ($CD5^-CD11b^-CD19^+$, **D**). The 3xTgAD mouse B cells, such as B1a (top panel), B1b (middle panel) and follicular B cells (FO, bottom panel, **E**-**G**), showed activated phenotype, as they upregulated expression of IFNγ (**E**), IL-6 (**F**), IL-10 (**G**) and TGFβ (**H**) in the cervical lymph nodes (CLN) as compared to WT mice. The AD markedly increased 4-$1BBL^+$ B1a, 4-$1BBL^+$ B1b and 4-$1BBL^+$ B2 cells in the spleen (**I**) and cervical lymph nodes (**J**). Shown is Mean ± SEM of cell numbers (**A**, **E**-**J**) and frequency (**B**-**D**) in spleen (**A**-**D** and **I**) and CLN (**E**-**H** and **J**), where each symbol is for a single mouse, n=4-6. Gating strategy is shown in Suppl. Fig. 9A. * $P < 0.05$; ** $P < 0.01$; *** $P < 0.001$ in unpaired *t*-test, except for **B** and **I** middle panel which are analyzed with Mann-Whitney test because the data are not distributed normally.

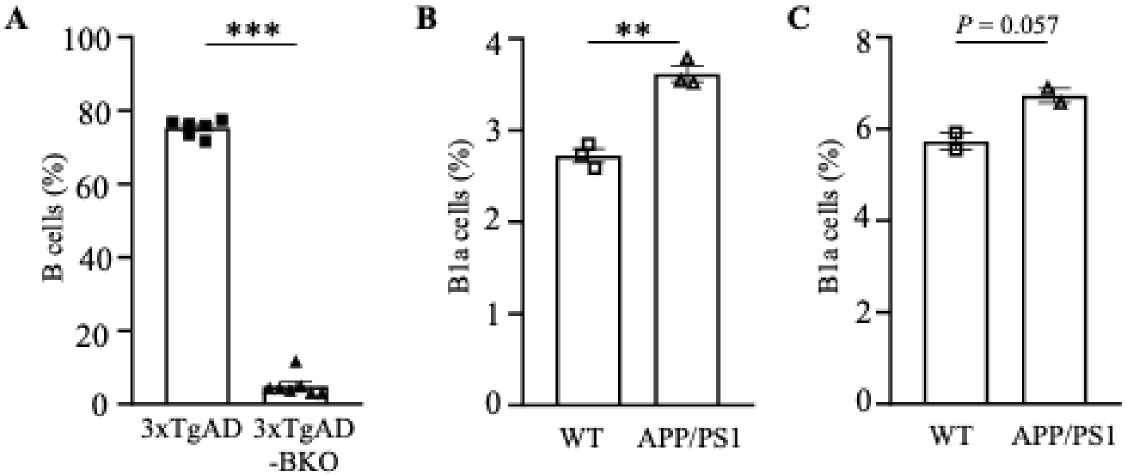

Supplementary Figure 2. B cells in the circulation of AD mice. 3xTgAD-BKO mice do not have circulating B cells (shown a representative data from peripheral blood, **A**). As in 3xTgAD mice, APP/PS1 mice also increased B1a cells in the blood (**B**) and cervical lymph nodes (**C**) as compared to age-and sex-matched WT littermates. Shown is Mean ± SEM, where each symbol is for a single mouse. Gating strategy is shown in Suppl. Fig. 9A.
** $P < 0.01$; *** $P < 0.001$ in unpaired *t*-test.

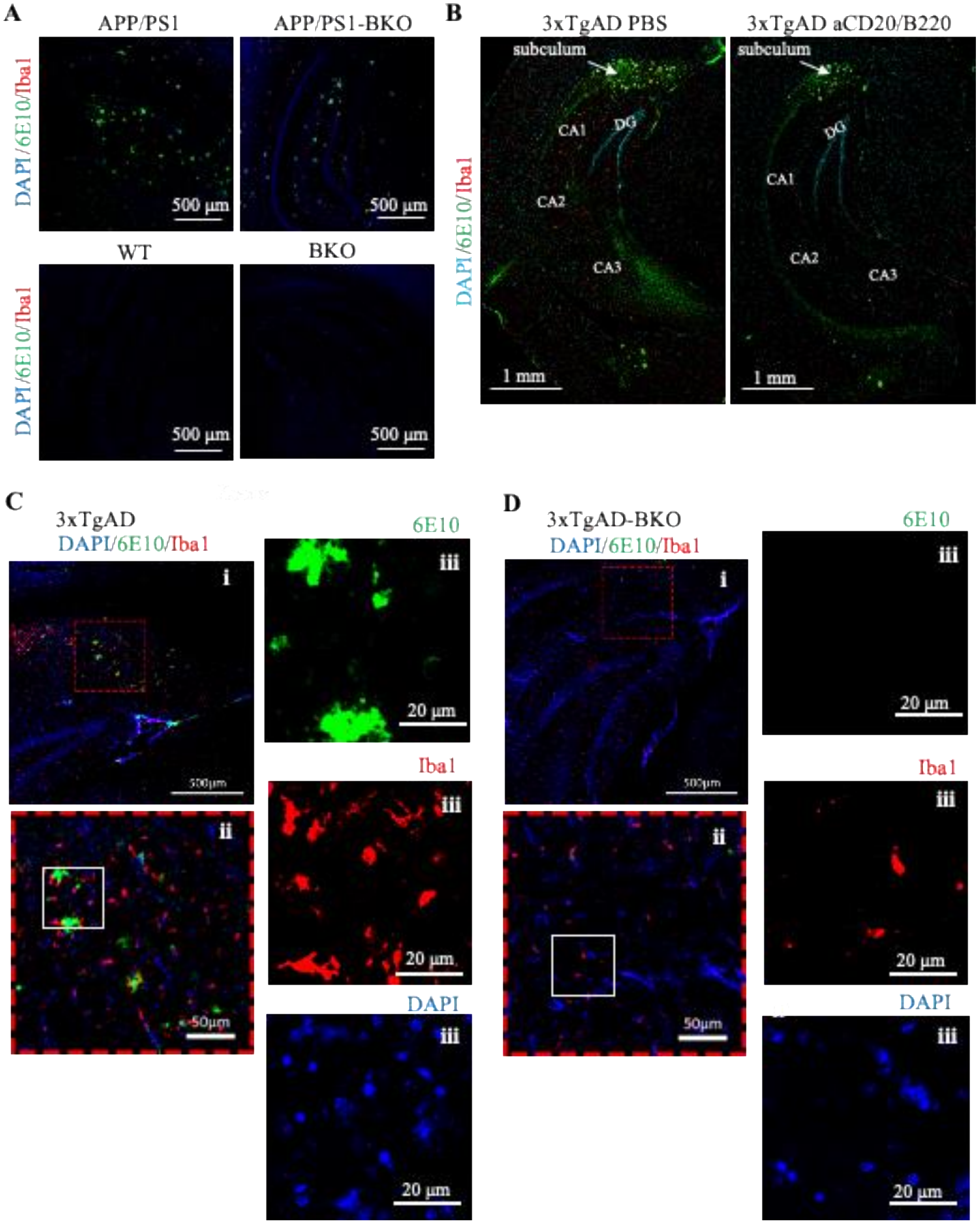
A
APP/PS1
APP/PS1-BKO
DAPI/6E10/Iba1
500 µm
500 µm
WT
BKO
DAPI/6E10/Iba1
500 µm
500 µm
B
3xTgAD PBS
3xTgAD aCD20/B220
subculum
CA1
DG
CA2
CA3
1 mm
DAPI/6E10/Iba1
C
3xTgAD
DAPI/6E10/Iba1
i
ii
6E10
iii
20 µm
Iba1
DAPI
D
3xTgAD-BKO
50µm

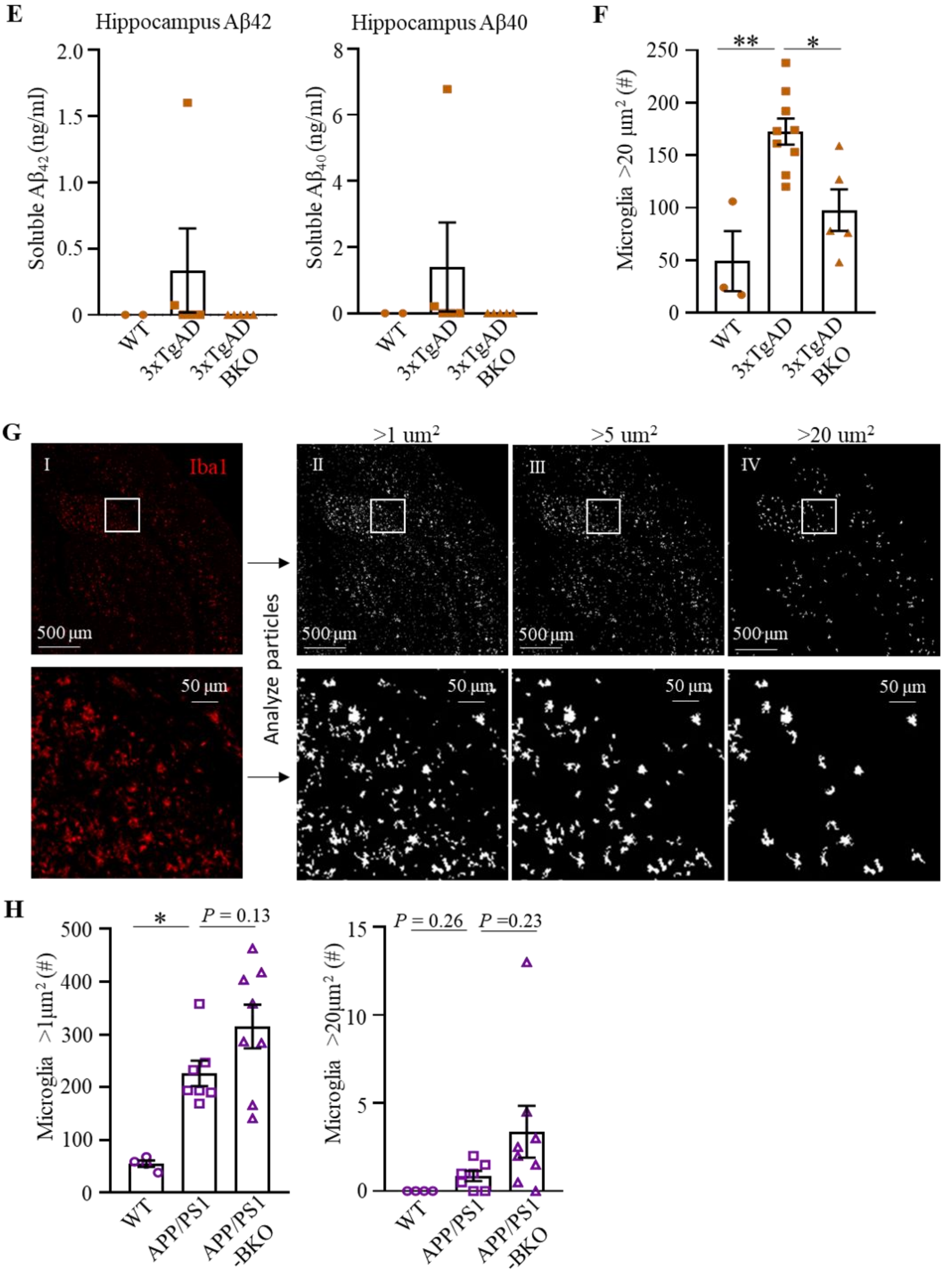
E
Hippocampus Aβ42
Soluble Aβ42 (ng/ml)
2.0
1.5
1.0
0.5
0
WT
3xTgAD
3xTgAD BKO
Hippocampus Aβ40
Soluble Aβ40 (ng/ml)
8
6
4
2
0
WT
3xTgAD
3xTgAD BKO
F
Microglia >20 µm² (#)
**
*
250
200
150
100
50
0
WT
3xTgAD
3xTgAD BKO
G
I
Iba1
500 µm
50 µm
Analyze particles
>1 um²
>5 um²
>20 um²
II
III
IV
500 µm
50 µm
H
Microglia >1µm² (#)
*
P = 0.13
500
400
300
200
100
0
WT
APP/PS1
APP/PS1 -BKO
Microglia >20µm² (#)
P = 0.26
P =0.23
15
10
5
0
WT
APP/PS1
APP/PS1 -BKO

Supplementary Figure 3. The loss of B cells reverses Aβ plaque burden and microglial activation in the hippocampus. Compared with APP/PS1 mice, APP/PS1-BKO mice markedly reduced Aβ plaques (green, scale, 500 μm, **A**). In 3xTgAD mice, both B-cell depletion with anti-CD20/B220 antibody **(B)** or genetic B-cell deficiency (**C**, **D**) decreased Aβ plaques in the hippocampus. Shown are Aβ plaques (green, **B**-**D**), $Iba1^+$ microglia in the subiculum (red, **C** and **D**), and DAPI (blue, **A**-**D**). Arrows in **B** show the subiculum area, where Aβ plaques and $Iba1^+$ microglia were quantified. In C and D, the enlarged images in red square (**i**) are shown in **ii** where white square is for the enlarged images in **iii**. The increased presence of soluble $A\beta_{1\text{-}40}$ and $A\beta_{1\text{-}42}$ peptides in the hippocampal lysates of 3xTgAD mice was lost in 3xTgAD-BKO mice (measured with ELISA, n=2-6, **E**). Larger-sized microglia (>20 μm^2) increased in the hippocampus of 3xTgAD mice as compared with age- and sex-matched 3xTgAD-BKO and WT mice (**F**). A representative strategy for quantification of microglia of different sizes is shown in **G** (see the Material and Methods section), the single channel of Iba1 image (**I**) were converted into 8-bit image, after proper threshold was set, "analyze particles" function was used for quantifying microglia with size above 1 μm^2 (**II**), 5 μm^2 (**III**) or 20 μm^2 (**IV**). The white squares are for enlarged images shown in lower panels. Unlike 3xTgAD mice, B-cell deficiency in APP/PS1 mice did not affect numbers of microglia (small size, >1 μm^2; and large size, >20 μm^2, **H**). Shown is Mean ± SEM (**E**,**F**, and **H**) * $P < 0.05$; ** $P < 0.01$ in one-way ANOVA (**F**, **H** left) or Kruskal-Wallis test (**H** right).

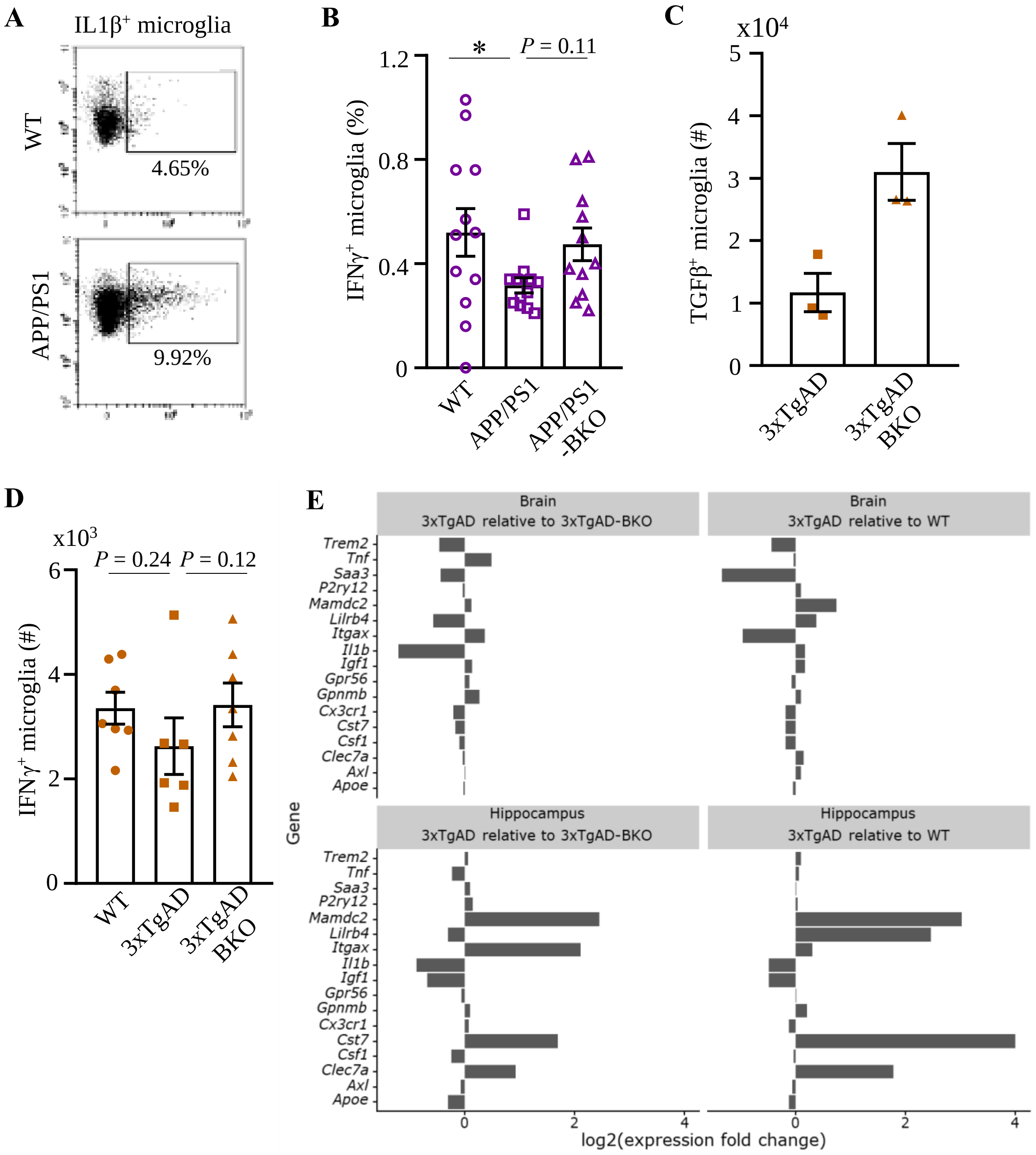

Supplementary Figure 4. B-cell deficiency restores resting microglia in AD mice. Representative dot plots show upregulation of IL1β in microglia (gated in $CD45^{Int}CD11b^{+}$) in the brain of female APP/PS1 mice as compared to WT mice (**A**). The B-cell deficiency in AD mice increased $IFN\gamma^{+}$ microglia in APP/PS1 mice (**B**) and $TGF\beta^{+}$ microglia in 3xTgAD mice (**C** and **D**). RNA microarray analyses revealed that the B-cell deficiency differentially affects expression of DAM-associated genes in the brain (left) and hippocampus (right panel, n=2-3). Shown is Mean ± SEM (**B-D**) and log2 fold-change in 3xTgAD as compared with 3xTgAD-BKO and WT, **E**). Gating strategy is shown in Suppl. Fig. 9B. * $P < 0.05$; in one-way ANOVA (**B**, **D**) or Mann-Whitney test (**C**).

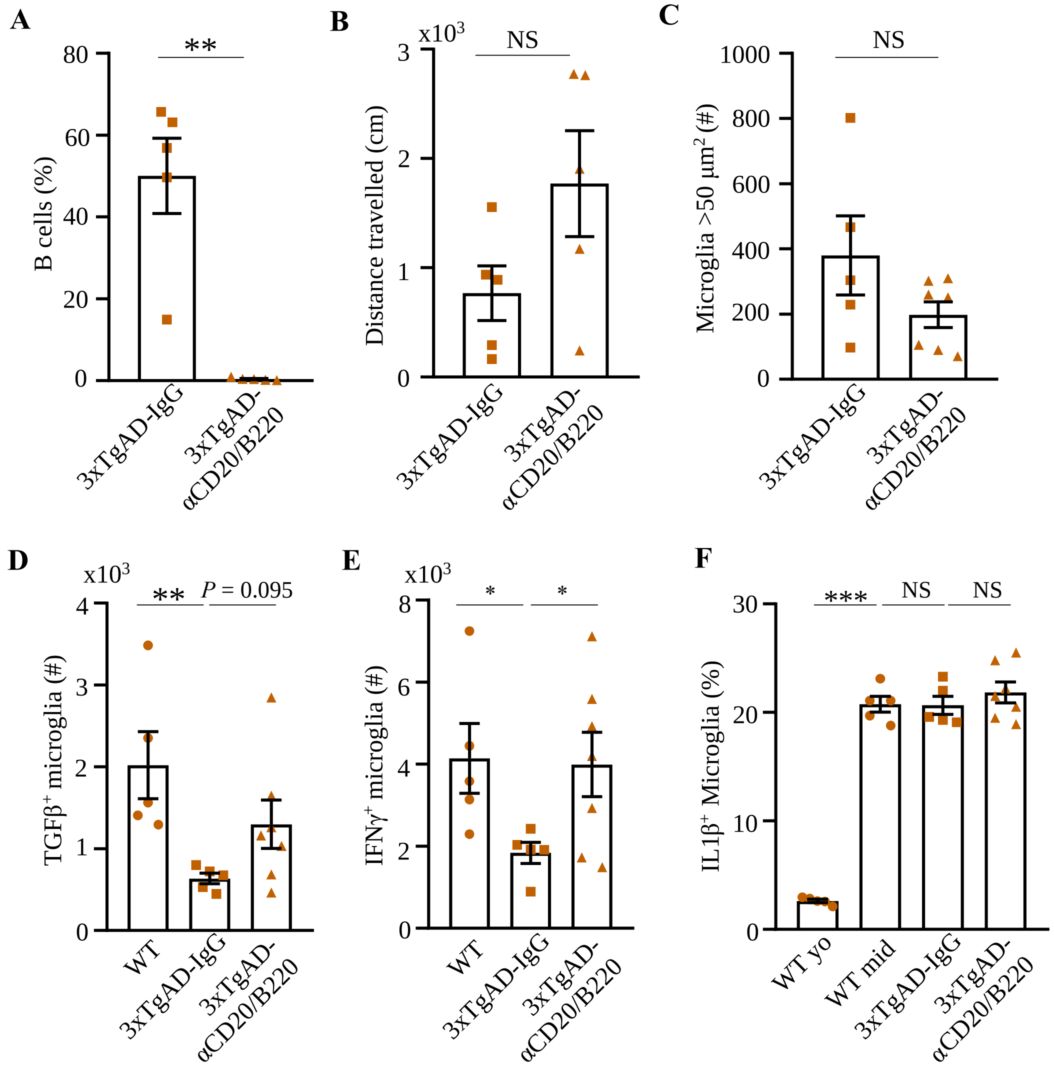

Supplementary Figure 5. The therapeutic benefits of B cell depletion in 3xTgADmice. A 2-month i.p. treatment with anti-CD20/B220 antibody significantly depleted B cells in the circulation of 3xTgAD mice (**A**). Controls were injected with isotype-matched IgG. Open field test revealed that the B-cell depletion improved their locomotion, as shown by the increase of the total distance travelled (in duration of 30 min, **B**). B-cell depletion did not affect total number of large-sized microglia (> 50 µm^2, **C**); however, it reversed the loss of TGFβ$^+$ (**D**) and IFNγ$^+$ (**E**) microglia in 3xTgAD mice without affecting IL1β expression (**F**). Shown is Mean ± SEM with each symbol is for a single mouse. Gating strategy is shown in Suppl. Fig. 9A, B. ** $P < 0.01$; *** $P < 0.001$ in Mann-Whitney test (**A**), unpaired *t*-test (**B**, **C**), Kruskal-Wallis test (**D**) or one-way ANOVA (**E**, **F**).

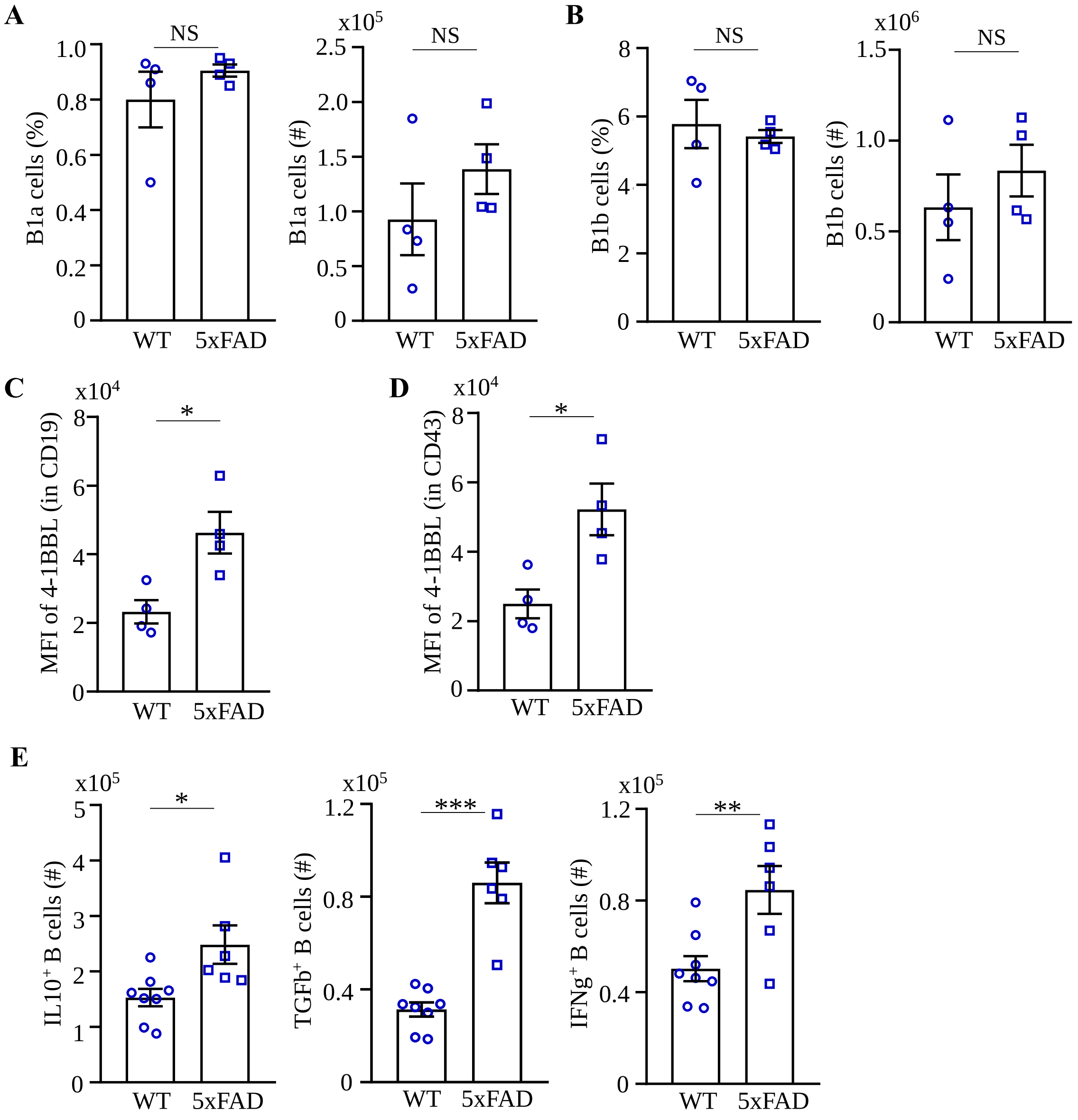

Supplementary Figure 6. B cells are activated in 5xFAD mice. Compared with age- and sex-matched WT mice, the frequency and the number of B1a (**A**) and B1b (**B**) cells are not changed in the spleen of 5xFAD mice. However, 5xFAD mice upregulated both B cells ($CD19^+$, C) and B1 cells ($CD43^+CD19^+$, **D**) expressing 4-1BBL (MFI) as compared with control WT mice. Moreover, 5xFAD mice upregulated B cells expressingIL10 (left), TGFβ (middle), and IFNγ (right panel, **E**), suggesting that AD activates B cells. Shown is Mean ± SEM with each symbol is for a single mouse. Gating strategy is shown in Suppl. Fig. 9A. * $P < 0.05$; ** $P < 0.01$; *** $P < 0.001$ in unpaired *t*-test.

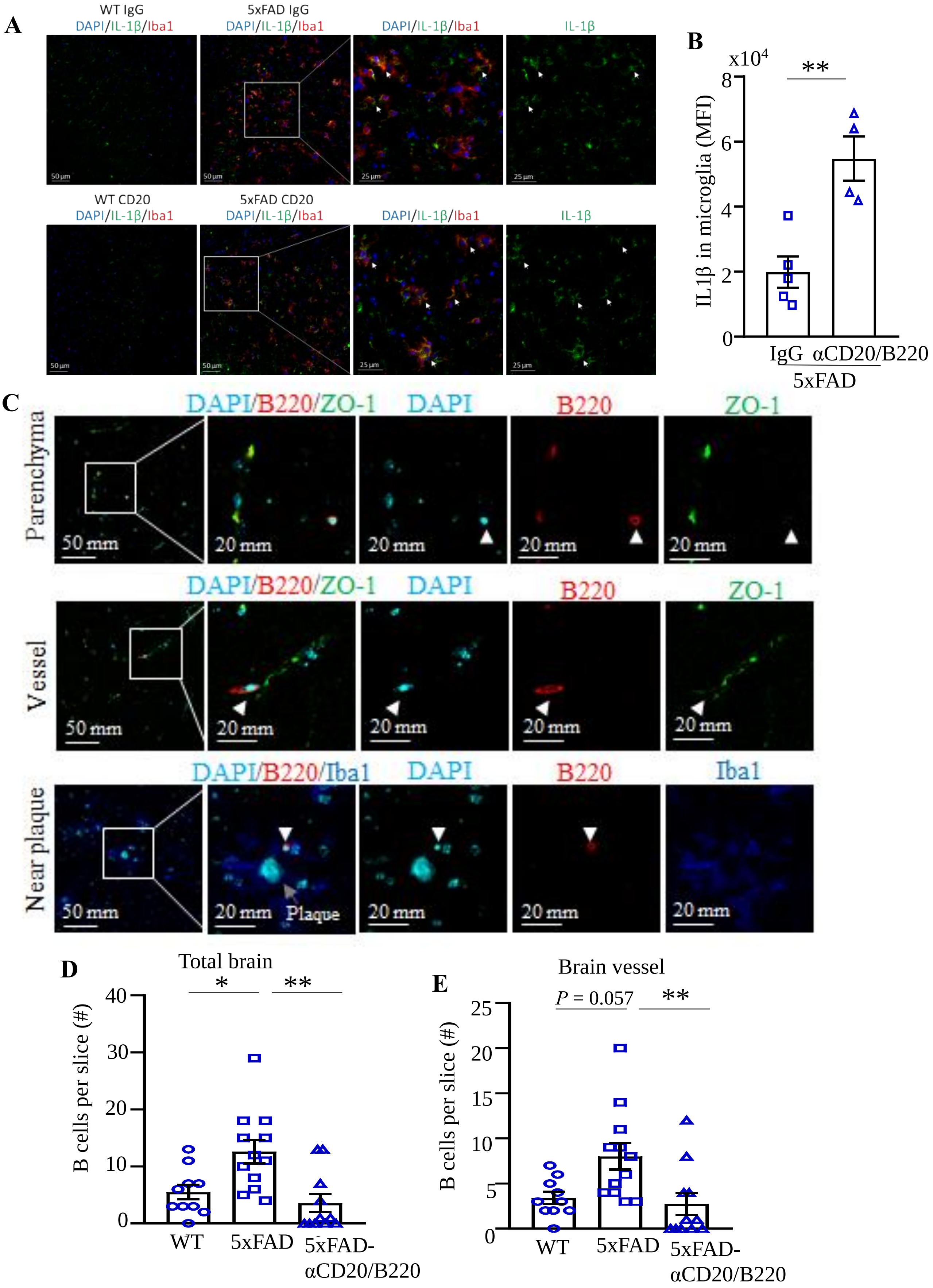
A
WT IgG
DAPI/IL-1β/Iba1
5xFAD IgG
DAPI/IL-1β/Iba1
DAPI/IL-1β/Iba1
IL-1β
WT CD20
DAPI/IL-1β/Iba1
5xFAD CD20
DAPI/IL-1β/Iba1
DAPI/IL-1β/Iba1
IL-1β
B
x10^4
**
IL1β in microglia (MFI)
0
2
4
6
8
IgG
αCD20/B220
5xFAD
C
Parenchyma
Vessel
Near plaque
DAPI/B220/ZO-1
DAPI
B220
ZO-1
DAPI/B220/Iba1
Iba1
50 mm
20 mm
Plaque
D
Total brain
*
**
B cells per slice (#)
0
10
20
30
40
WT
5xFAD
5xFAD-
αCD20/B220
E
Brain vessel
P = 0.057
**
B cells per slice (#)
0
5
10
15
20
25
WT
5xFAD
5xFAD-
αCD20/B220

Supplementary Figure 7. Immunofluorescent staining of IL1β$^+$ microglia and B220$^+$ B cells in the brain of female 5xFAD mice. The representative image of IL1β expression in the subiculum (IL1β, green; and Iba1$^+$ microglia, red; the arrow heads are for IL1β co-localized with Iba1$^+$ microglia, **A**) and the quantification of IL1β intensity in Iba1$^+$ microglia (n=4-5, **B**). B cells were found markedly increased in the brain parenchyma of 5xFAD mice as compared with WT mice (**C**). Immune fluorescent staining shows B220$^+$ B cells in the brain parenchyma (upper panels), within vessels (middle panels) and near plaques (bottom panels, **C**). Quantification of B220$^+$ B cells in the whole brain parenchyma and in the brain vessels is in **D** and **E**, respectively (n=10-12). In B, D and E, shown is Mean ± SEM; each symbol is for a single mouse. * $P < 0.05$; ** $P < 0.01$ in unpaired *t*-test (**B**) or Kruskal-Wallis test (**D**-**E**).

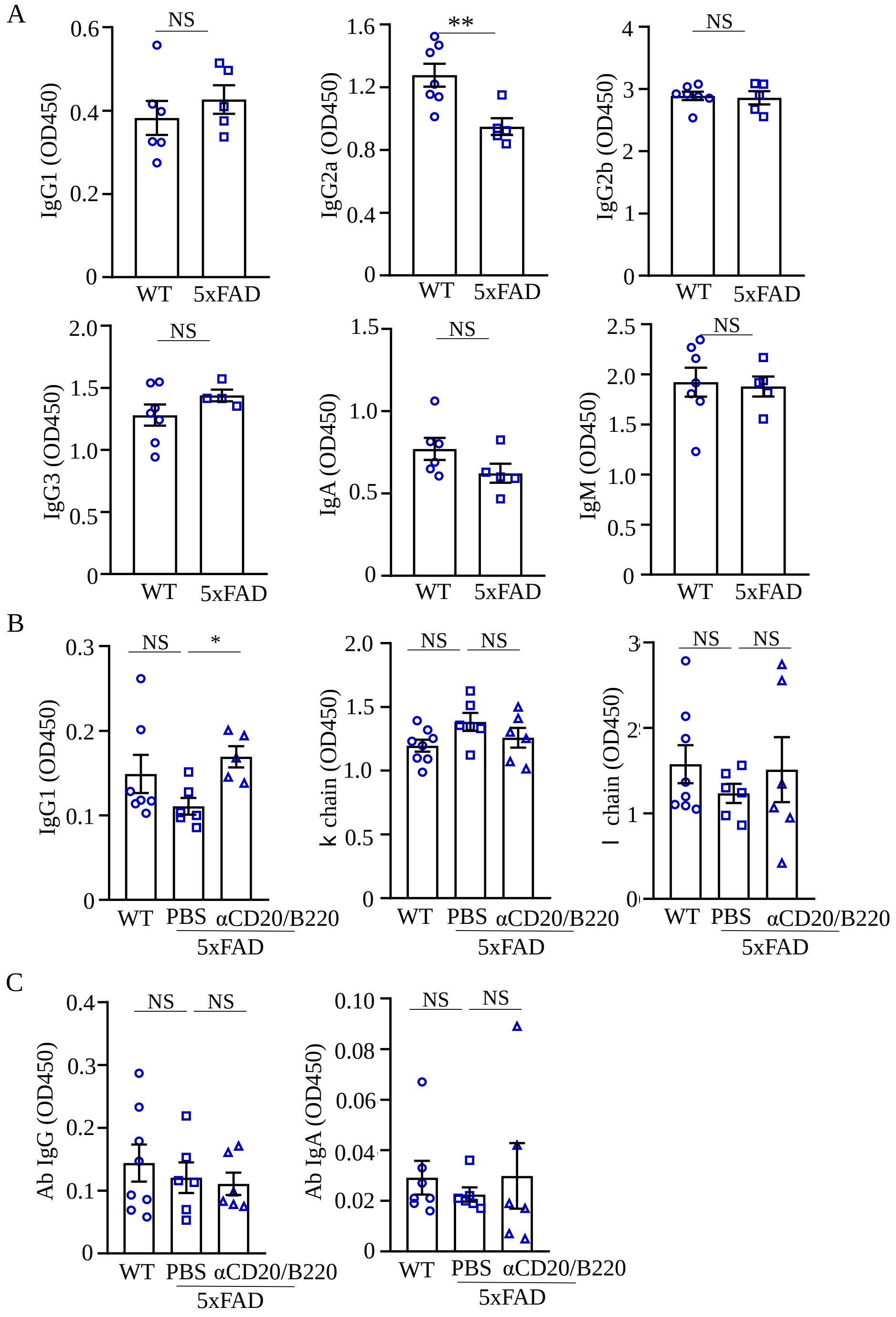

Supplementary Figure 8. Transient B-cell depletion in 5xFAD mice does not affect the levels of immunoglobulin in circulation. In A, shown is ELISA quantification of IgG1, IgG2a, IgG2b, IgG3, IgA and IgM in sera of 5xFAD mice as compared with WT mice. Compared with control, a 2-month B-cell depletion (5xFAD-aCD20/B220) did not affect serum levels of IgG1, κ chain and λ chain Igs (**B**). 5xFAD did not have a tangible amount of Aβ-specific antibody in the circulation (**C**). Shown is Mean ± SEM; each symbol is for a single mouse. Statistical analyses are conducted with unpaired *t*-test (**A**), Kruskal-Wallis test (**B** left) or one-way ANOVA (middle and right in **B**, and **C**).

A

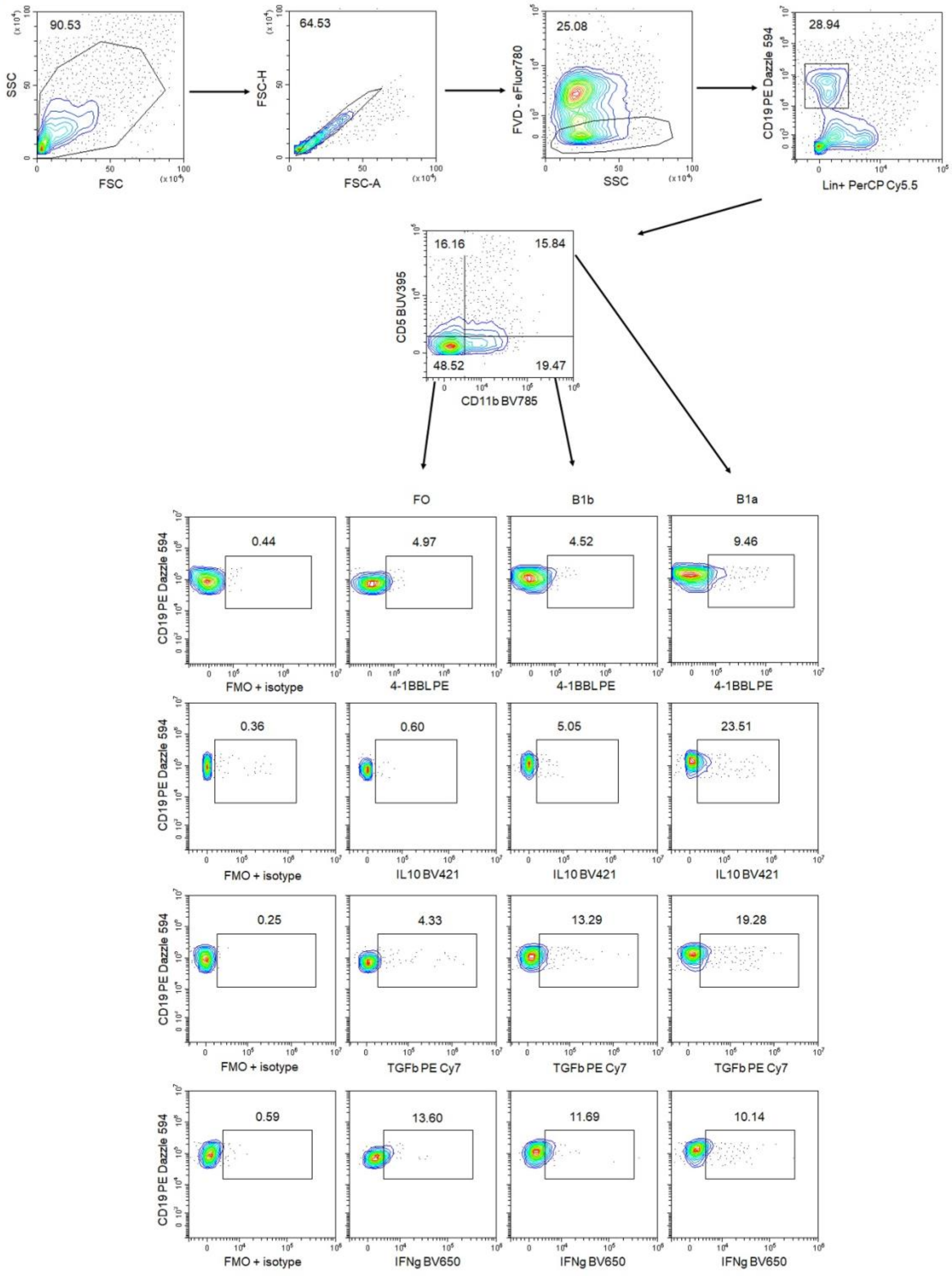

90.53
SSC
FSC
64.53
FSC-H
FSC-A
25.08
FVD - eFluor780
SSC
28.94
CD19 PE Dazzle 594
Lin+ PerCP Cy5.5
16.16
15.84
48.52
19.47
CD5 BUV395
CD11b BV785
FO
B1b
B1a
0.44
4.97
4.52
9.46
FMO + isotype
4-1BBL PE
0.36
0.60
5.05
23.51
IL10 BV421
0.25
4.33
13.29
19.28
TGFb PE Cy7
0.59
13.60
11.69
10.14
IFNg BV650

B

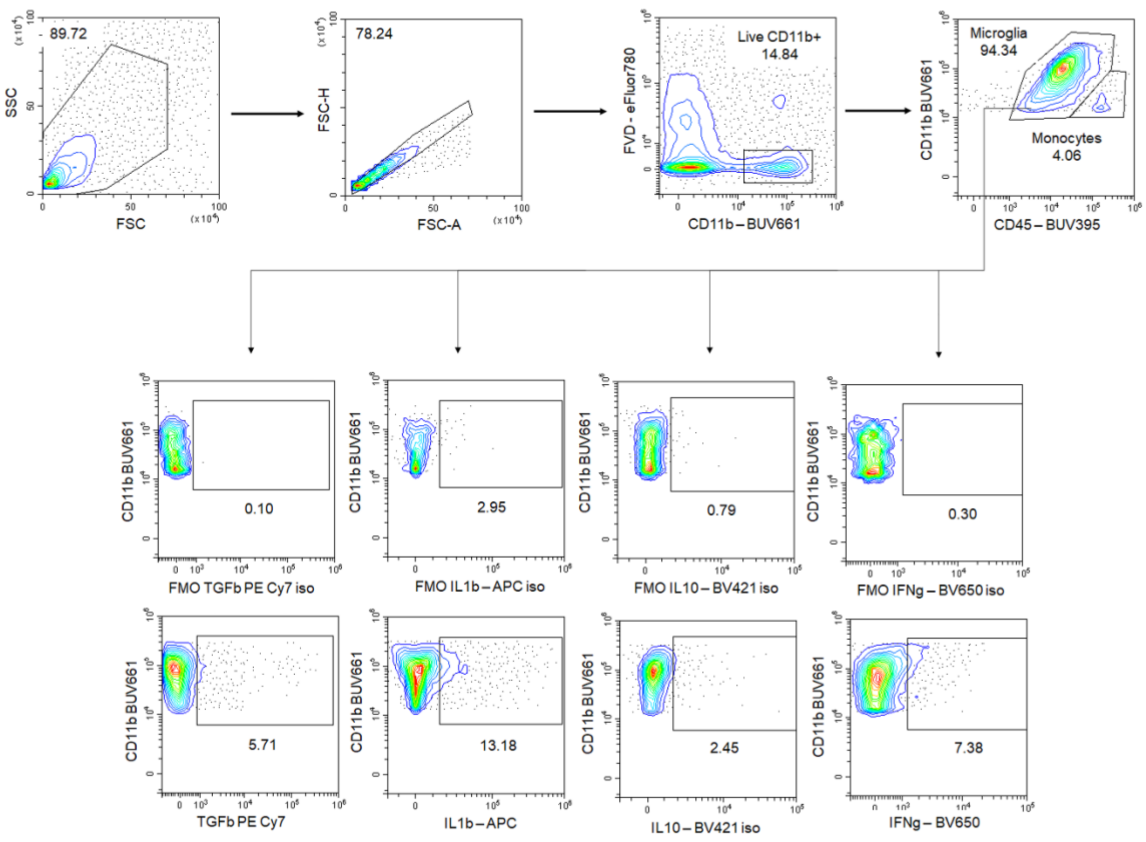

Supplementary Figure 9. Gating strategy used in the study to determine the percentage of cytokine expressing B-cell subsets (**A,** depicted in Fig. 1A-G, Fig. 2C, Suppl. Fig. 1A-J, Suppl. Fig. 2A-C, Suppl. Fig. 5A, Suppl. Fig. 6A-E) and microglia **(B,** depicted in Fig. 4A-C, G; Fig. 5E-F; Suppl. Fig. 4A-D and Suppl. Fig. 5D-F**)**.
