# Supplementary material for: Therapeutic B-cell depletion reverses progression of Alzheimer’s disease
Source: Nat Commun. 2021 Apr 12;12:2185. doi: 10.1038/s41467-021-22479-4 (PMC8042032; doi:10.1038/s41467-021-22479-4)
Supplement: Supplementary file 3 — Description of Additional Supplementary Files [file 41467_2021_22479_MOESM3_ESM.pdf]

Description of Additional Supplementary Files

File Name: Supplementary Data 1
Description: List of AD-related gene expression level from total mouse brain

File Name: Supplementary Data 2
Description: Top 100 genes changed in hippocampus of 3xTgAD vs 3xTgAD-BKO, female

File Name: Supplementary Data 3
Description: Top 100 genes changed in brain of 3xTgAD vs 3xTgAD-BKO, female

File Name: Supplementary Data 4
Description: List of antibody used in flow cytometry analysis
