# Supplementary material for: Therapeutic B-cell depletion reverses progression of Alzheimer’s disease
Source: Nat Commun. 2021 Apr 12;12:2185. doi: 10.1038/s41467-021-22479-4 (PMC8042032; doi:10.1038/s41467-021-22479-4)
Supplement: Supplementary file 8 — Reporting Summary [file 41467_2021_22479_MOESM8_ESM.pdf]

nature research

Corresponding author(s): Biragyn; NCOMMS-19-40031B

Last updated by author(s): Feb 2, 2021

Reporting Summary

Nature Research wishes to improve the reproducibility of the work that we publish. This form provides structure for consistency and transparency in reporting. For further information on Nature Research policies, see our Editorial Policies and the Editorial Policy Checklist.

Statistics

For all statistical analyses, confirm that the following items are present in the figure legend, table legend, main text, or Methods section.

n/a	Confirmed	
☐	☒	The exact sample size (*n*) for each experimental group/condition, given as a discrete number and unit of measurement
☒	☐	A statement on whether measurements were taken from distinct samples or whether the same sample was measured repeatedly
☐	☒	The statistical test(s) used AND whether they are one- or two-sided<br>*Only common tests should be described solely by name; describe more complex techniques in the Methods section.*
☒	☐	A description of all covariates tested
☒	☐	A description of any assumptions or corrections, such as tests of normality and adjustment for multiple comparisons
☐	☒	A full description of the statistical parameters including central tendency (e.g. means) or other basic estimates (e.g. regression coefficient) AND variation (e.g. standard deviation) or associated estimates of uncertainty (e.g. confidence intervals)
☐	☒	For null hypothesis testing, the test statistic (e.g. *F*, *t*, *r*) with confidence intervals, effect sizes, degrees of freedom and *P* value noted<br>*Give P values as exact values whenever suitable.*
☒	☐	For Bayesian analysis, information on the choice of priors and Markov chain Monte Carlo settings
☒	☐	For hierarchical and complex designs, identification of the appropriate level for tests and full reporting of outcomes
☒	☐	Estimates of effect sizes (e.g. Cohen's *d*, Pearson's *r*), indicating how they were calculated

Our web collection on statistics for biologists contains articles on many of the points above.

Software and code

Policy information about availability of computer code

Data collection	Data were analyzed on FACS Canto II (BD) or CytoFLEX (Beckman Coulter, Inc.)
Data analysis	Software used "GraphPad Prism (Prism 8; Graph Pad Software, Inc." is noted In Methods section

For manuscripts utilizing custom algorithms or software that are central to the research but not yet described in published literature, software must be made available to editors and reviewers. We strongly encourage code deposition in a community repository (e.g. GitHub). See the Nature Research guidelines for submitting code & software for further information.

Data

Policy information about availability of data

All manuscripts must include a data availability statement. This statement should provide the following information, where applicable:

- Accession codes, unique identifiers, or web links for publicly available datasets
- A list of figures that have associated raw data
- A description of any restrictions on data availability

Microarray data are submitted at https://www.ncbi.nlm.nih.gov/geo/query/acc.cgi?acc=GSE165111

Field-specific reporting

Please select the one below that is the best fit for your research. If you are not sure, read the appropriate sections before making your selection.

- [x] Life sciences
- [ ] Behavioural & social sciences
- [ ] Ecological, evolutionary & environmental sciences

For a reference copy of the document with all sections, see nature.com/documents/nr-reporting-summary-flat.pdf

Life sciences study design

All studies must disclose on these points even when the disclosure is negative.

Sample size	not perfrmed
Data exclusions	no
Replication	Data were independently reproduced at least twice
Randomization	All samples were randomized based on age and sex of mice
Blinding	Samples/mice were analyzed based on mouse ID #. Investigators were blinded during data collection and analyses

Reporting for specific materials, systems and methods

We require information from authors about some types of materials, experimental systems and methods used in many studies. Here, indicate whether each material, system or method listed is relevant to your study. If you are not sure if a list item applies to your research, read the appropriate section before selecting a response.

Materials & experimental systems

n/a	Involved in the study
☐	☒ Antibodies
☒	☐ Eukaryotic cell lines
☒	☐ Palaeontology and archaeology
☐	☒ Animals and other organisms
☒	☐ Human research participants
☒	☐ Clinical data
☒	☐ Dual use research of concern

Methods

n/a	Involved in the study
☒	☐ ChIP-seq
☐	☒ Flow cytometry
☒	☐ MRI-based neuroimaging

Antibodies

Antibodies used	Antibodies used are described in the Material/Methods section and also in Suppl. Table 4
Validation	Antibodies used are described in the Material/Methods section and also in Suppl. Table 4. Antibody validation was done based on preliminary stainings in comparison with isotype-matched control antibodies. Whenever relevant, we also included references to our other papers where we used the antibodies.

Animals and other organisms

Policy information about studies involving animals; ARRIVE guidelines recommended for reporting animal research

Laboratory animals	Mouse strain, sex, age is described in the Material/Method and in the Results section. 3xTgAD mice and their litermate controls were 60-70 weeks female mice. APP/PS1 and 5xFAD mice and their litermate controls were 20-35 weeks of age female and male mice. AD transgenic mice were bred, aged and housed in housed in the same , SPF environment at NIA, while sex- and age- matched WT mice (C57BL/c) were purchased from Jackson Laboratory (Bar Harbor, ME) and housed in the same room (as AD transgenic mice) for 1-2 weeks before their use in experiments.
Wild animals	None
Field-collected samples	None
Ethics oversight	Used approved protocol 321-LMBI-2022 by NIA ACUC committee of the National Institute on Aging

Note that full information on the approval of the study protocol must also be provided in the manuscript.

Flow Cytometry

Plots

Confirm that:

☒ The axis labels state the marker and fluorochrome used (e.g. CD4-FITC).

☒ The axis scales are clearly visible. Include numbers along axes only for bottom left plot of group (a 'group' is an analysis of identical markers).

☒ All plots are contour plots with outliers or pseudocolor plots.

☒ A numerical value for number of cells or percentage (with statistics) is provided.

Methodology

Sample preparation	Single cell suspension of spleen and cervical lymph node (cLN) was prepared using a 70 µm cell strainer (BD Falcon, Bedford, MA).
Instrument	Data were analyzed on FACS Canto II (BD) or CytoFLEX (Beckman Coulter, Inc.)
Software	FlowJo software (Tree Star, Inc.) or CytExpert software (Beckman Coulter, Inc.).
Cell population abundance	Brain B cells represented <1% of CD45+ cells.
Gating strategy	For FACS, life cells and single cell population was gated. Gating for positive cells was set based on staining of isotype-matched negative control antibody conjugated with the same fluorochrome.

☒ Tick this box to confirm that a figure exemplifying the gating strategy is provided in the Supplementary Information.
